# The BCG World Atlas: A Database of Global BCG Vaccination Policies and Practices

**DOI:** 10.1371/journal.pmed.1001012

**Published:** 2011-03-22

**Authors:** Alice Zwerling, Marcel A. Behr, Aman Verma, Timothy F. Brewer, Dick Menzies, Madhukar Pai

**Affiliations:** 1 Department of Epidemiology, Biostatistics & Occupational Health, McGill University, Montreal, Canada; 2 Respiratory Epidemiology & Clinical Research Unit, Montreal Chest Institute, Montreal, Canada; 3 Department of Medicine, McGill University Health Centre, Montreal, Canada; 4 Global Health Programs, Faculty of Medicine, McGill University, Montreal, Canada

## Abstract

Madhu Pai and colleagues introduce the BCG World Atlas, an open access, user friendly Web site for TB clinicians to discern global BCG vaccination policies and practices and improve the care of their patients.

**Summary Points**

- Despite nearly a century of use, the Bacille Calmette-Guérin (BCG) vaccine continues to be controversial, with known variations in BCG substrains and vaccine efficacy.
- Because vaccination policies and practices vary across time and countries, we created the first searchable, online, open access database of global BCG vaccination policy and practices, the BCG World Atlas (http://www.bcgatlas.org/), which contains detailed information on current and past BCG policies and practices for over 180 countries.
- The Atlas is for clinicians, policymakers, and researchers and provides information that may be helpful for better interpretation of tuberculosis (TB) diagnostics as well as design of new TB vaccines.

## Tuberculosis: A Global Threat

Tuberculosis (TB) remains one of the major causes of infectious morbidity and mortality globally, claiming millions of lives every year. Approximately one-third of the world's population is estimated to be infected with *Mycobacterium tuberculosis*, giving rise to 9.4 million new cases of active TB disease each year [1]. The majority of the TB burden exists in 22 high-burden countries, but with immigration and global travel TB is difficult to eliminate from any one country [1]–[4].

The Bacille Calmette-Guérin (BCG) vaccine, first introduced in 1921, continues to be the only vaccine used to prevent TB [5],[6]. Despite nearly a century of use, BCG remains controversial, with known variations in BCG substrains, vaccine efficacy, policies, and practices across the world. Global information on BCG policies and practices may be useful for clinical interpretation of diagnostic tests as well as in the design of novel TB vaccines that are under development.

## BCG: A Range of Global Policies

While most experts agree that BCG is efficacious against severe forms of childhood TB, its efficacy against TB in adults is highly variable [7]. As a result of the uncertain efficacy of the BCG vaccine, countries have developed very different BCG vaccination policies. Some countries, such as the United Kingdom, have or have had universal BCG vaccination programs, while others (including Canada and the United States) either only recommended BCG for high-risk groups or did not advocate BCG countrywide. The Canadian situation was further complicated by differing policies across provinces, where some provinces underwent mass vaccination programs and others did not. In addition, BCG vaccination policies have varied by the number of doses used, the age at which vaccination was given, and the methods used to deliver the vaccine (although most countries today use only the intradermal route) [8]. Vaccination practices also have changed within and across countries over the years, reflecting changes in evidence, health policy, public perception, increasing or decreasing TB incidence, and HIV incidence. As a result of these changes to the BCG policies in various countries, it is necessary to not only know the current BCG vaccination policies but also past policies and applicable changes when dealing with adults who received BCG vaccination in childhood.

Since the publication of the *M. tuberculosis* genome, comparative genomic studies have documented that BCG vaccine strains have evolved and differ from each other and from the original BCG first used in 1921 [9]. Because these genetic differences affect antigenic proteins, these changes may translate into differences in efficacy and effect on the tuberculin skin test (TST) [10],[11]. Work done by Ritz and Curtis looking at global BCG strain variations demonstrates the diversity of strains used by different countries and even within the same countries [12]. They found that 44% (83/188) of countries reported using more than one BCG strain type during an interval of only 5 years. This highlights the importance of documenting BCG vaccination practices for both clinical and research purposes.

**Figure 1:**
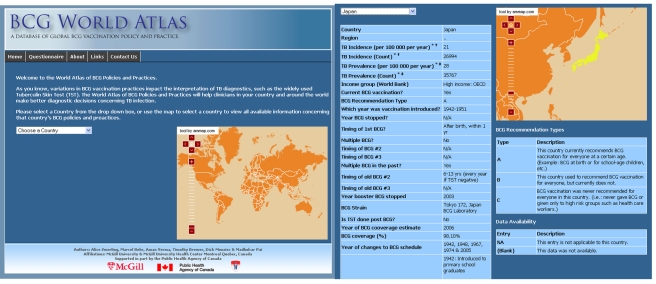
Home page of the BCG World Atlas (http://www.bcgatlas.org/) and example of BCG policies and practices in Japan.

Clinicians cannot be expected to know BCG practices in all countries, and immigrants themselves may not know about the vaccination policies in their countries of birth (most adult immigrants are unlikely to retain childhood vaccination records). Information on diversity of BCG policies between countries and across time may be helpful for better interpretation of TB diagnostics as well as design of new TB vaccines. To our knowledge, there is no single, comprehensive, searchable database of BCG policies and practices (past and present) across the world. We developed the “BCG World Atlas: A Database of Global BCG Vaccination Policies and Practices,” a database containing BCG information from each country across all world regions (http://www.bcgatlas.org/). Figure 1 shows the homepage of the Atlas. To make this resource practical and useful for clinicians, public health practitioners, and researchers alike, our database captures both present and previous policy and practices within a country, as well as any applicable changes.

## Assembling the Database

Detailed information on past and present BCG vaccination policies and practices were collected from as many countries as possible by one of three methods. First, short respondent-completed questionnaires were sent out to at least two individuals in each country. Questionnaires were sent to experts in TB research, TB control programs, or public health/vaccination programs. Whenever possible, an attempt was made to collect two completed questionnaires from each country in order to validate the data. Questionnaires were available in English, French, and Spanish, and were designed to capture both current policy and actual BCG practices, as well any applicable changes that had occurred over the last 25 years. Detailed questions asked for information concerning past as well as current practices, the timing and nature of changes to the policies and or practices, repeat, multiple, or booster shots, information concerning tuberculin skin testing in conjunction with BCG vaccination, influence of HIV on the decision to vaccinate, and vaccine strain differences. A total of 89 completed questionnaires were received over a 2-year period. Second, data were abstracted from published papers, reports, and available government policy documents retrieved through literature searches on PubMed and via the World Wide Web. Third, we used immunization data available from the World Health Organization Vaccine Preventable Diseases Monitoring System (http://apps.who.int/immunization_monitoring/en/globalsummary/ScheduleSelect.cfm), which provide basic information on all vaccines currently in use in each country [13].

Based on the data generated by all methods, countries were grouped into three main categories: A), the country currently recommends universal BCG vaccination at a certain age; B), the country used to recommend universal BCG vaccination but currently does not; or category C), BCG vaccination is recommended only for selected high-risk groups or was never recommended.

## Summary of Findings

**Figure 2:**
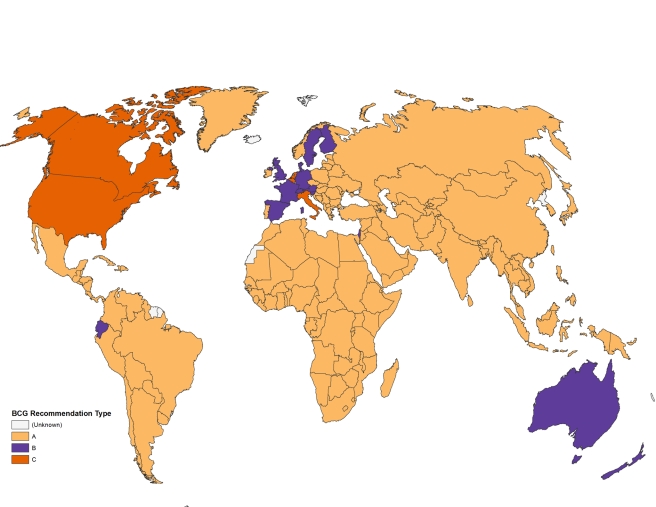
Map displaying BCG vaccination policy by country. A: The country currently has universal BCG vaccination program. B: The country used to recommend BCG vaccination for everyone, but currently does not. C: The country never had universal BCG vaccination programs.

The beta version of the Atlas went live in the fall of 2008 with completed questionnaires on BCG vaccination from 62 countries. Since that time, more data have been added and several improvements have been made. As of October 2010, we have collected data concerning BCG vaccination policies and practices for 180 of 209 (86%) countries worldwide that we approached. The database is available as an interactive Web site at http://www.bcgatlas.org/, where information for a particular country's BCG policy, along with its estimated World Health Organization (WHO) TB incidence statistics, can be viewed alongside a graphical map. Among the 180 countries with available data, 157 countries currently recommend universal BCG vaccination, while the remaining 23 countries have either stopped BCG vaccination (due to a reduction in TB incidence), or never recommended mass BCG immunization and instead favored selective vaccination of “at risk” groups (Figure 2). Complete questionnaire data were available for 77 countries, while remaining data were extracted from published sources.

Many countries began BCG vaccination programs in the 1940s–1980s, though some countries such as Romania and Uzbekistan report vaccination campaigns as early as 1928 and 1937, respectively, while some sub-Saharan African nations such as Nigeria and Sierra Leone only began BCG vaccinations in 1991 and 1990. Nine countries have ceased universal BCG vaccination programs; Spain and Denmark were among the first, stopping in 1981 and 1986, respectively, while Austria and Germany had stopped by 1990 and 1998. The remaining countries, including the Isle of Man, Slovenia, UK, Finland, and France, all ceased their BCG vaccination campaigns between 2005 and 2007. While these countries may have ceased mass universal vaccination programs, many do continue to provide BCG vaccination selectively to high-risk individuals, including those involved in high TB risk occupations and/or travel, and infants born into high TB risk environments.

We identified 49 countries that reported changes to their BCG vaccination policy in the past 20 years. Twenty-seven countries reported major changes to their BCG policy within the last 10 years. Of particular interest is the large number (*n* = 33) of countries that had multiple vaccination programs in the past, but have since ceased revaccination, and now use a single BCG vaccination schedule (Table 1). These revaccination policy changes were as recent as 2007. Sixteen countries continue to give an additional BCG vaccination after the initial BCG, known as a booster vaccination (Table 2), while Kazakhstan, Belarus, Uzbekistan, and Turkmenistan continue to recommend three BCG vaccinations, with the third given between the ages of 12 and 15. Multiple revaccinations may lead to a delayed hypersensitivity reaction also known as the Koch response (phenomenon), where a person previously infected with *M. tuberculosis* is reinfected intracutaneously, resulting in a local inflammatory reaction marked by necrotic lesions that develops rapidly and heals quickly [14].

**Table 1:** Countries that have ceased booster BCG vaccinations (*n* = 33).

Country	Current BCG Vaccination	Currently Recommend a Booster BCG	Booster BCG given in past?	Timing of old BCG boosters	Year Booster BCG stopped
Argentina	Yes	No	Yes	6 & 16 yrs	1995 & 2007
Bosnia and Herzegovina	Yes	No	Yes	3, 5, 7, 13, & 19 yrs	1996
Brazil	Yes	No	Yes	6 yrs	2006
Bulgaria	Yes	No	Yes	7 months, 7, 11, & 17 yrs	Unknown
Chile	Yes	No	Yes	5 yrs	2004
China	Yes	No	Yes	Unknown	Unknown
Estonia	Yes	No	Yes	7 yrs	2003
Finland	No	No	Yes	School-age (if TST negative)	1990
France	No	No	Yes	Unknown	Unknown
Hong Kong, China	Yes	No	Yes	Primary school	2000
Iran, Islamic Rep.	Yes	No	Yes	4–6 yrs	1999
Ireland	Yes	No	Yes	11–12 yrs	1996
Italy	No	No	Yes	9 yrs	2001
Japan	Yes	No	Yes	6–13 yrs (every year if TST negative)	2003
Korea, Rep.	Yes	No	Yes	8 yrs	2007
Latvia	Yes	No	Yes	7, 12, & 15 yrs	1974: (12 yrs ceased), 1993: (7 yrs ceased), 1998: Ceased all revaccination
Macedonia, FYR	Yes	Yes: Age: 7	Yes	14 yrs	Unknown
Mexico	Yes	No	Yes	6 yrs (school age)	1998
Mongolia	Yes	No	Yes	8, 15, & 18 yrs	1974–2006
Poland	Yes	No	Yes	7 yrs	2005
Romania	Yes	No	Yes	7, 14, & 18/21 yrs	1992–1993
Serbia and Montenegro	Yes	No	Yes	2, 7, 10, & 14 yrs	1997
Singapore	Yes	No	Yes	12 or 16 yrs	2001
Slovak Republic	Yes	No	Yes	7 & 14 yrs	2001
Slovenia	No	No	Yes	14–15 yrs	1947–1996
South Africa	Yes	No	Yes	Unknown	Unknown
Sweden	No	No	Yes	7 & 15 yrs (if non-reactive to TST)	1965: stopped booster at 7 yrs, 1986: stopped booster at 15 yrs, 1979: stopped boosters for military conscripts
Taiwan	Yes	No	Yes	12 yrs	1982–1997
Thailand	Yes	No	Yes	Unknown	Unknown
Turkey	Yes	No	Yes	7, 14, & 20 yrs	1997: stopped boosters at 14 & 20 yrs, 2006: stopped boosters at 7 yrs
Uruguay	Yes	No	Yes	6–12 yrs	1980–1993
Uzbekistan	Yes	Yes: Ages 7 & 14	Yes	12, 15, 20, 25, & 30 yrs	1997
Zambia	Yes	No	Yes	Unknown	Unknown

**Table 2:** Countries that currently recommend multiple BCG vaccinations (*n* = 16).

Country	Age of 1st BCG	Age of 2nd BCG	Age of 3rd BCG
Armenia	At birth	7 yrs, if no scar	-
Belarus	After birth, within 1 yr	7 yrs	14 yrs
Croatia	At birth	14 yrs	-
Czech Republic	At birth	11 yrs	-
Fiji	At birth	6 yrs	-
Kazakhstan	At birth	6 yrs	12 yrs
Macedonia, FYR	At birth	7 yrs	-
Moldova	After birth, within 1 yr	6–7 yrs	-
Nigeria	At birth	5 yrs (but not yet incorporated into the immunization schedule)	-
Norway	At birth	13–15 yrs	-
Philippines	At birth	Not specified	-
Russian Federation	At birth	7–14 yrs	-
Tunisia	At birth	6 yrs	-
Turkmenistan	After birth, within 1 yr	6–7 yrs	15 yrs
Ukraine	After birth, within 1 yr	7 yrs	-
Uzbekistan	At birth	7 yrs	14 yrs

Changes in vaccine strain were the most frequent type of change reported (*n* = 42), and many countries have employed several strains over the course of their BCG vaccination program's existence, with countries reporting strain changes as recently as 2008.

Additional variations in BCG vaccination administration are seen across countries. Currently, eight countries recommend TST post–BCG vaccination, and two other countries had this policy but have since ceased. Estimated national BCG coverage ranged from 70% to 100%; however, frequently these estimates were not available or were several years out of date. Finally, 19 countries that did not recommend universal BCG vaccination did report BCG vaccination for certain at-risk groups, most frequently health care workers and infants living in high-risk TB settings. These variations highlight the importance of mapping these differences across regions both for clinical purposes and research.

## Open Access through an Interactive Web Site

The Atlas is an interactive Web site that allows users to select and view information concerning a country's past and current BCG vaccination policy either by clicking on an interactive map or by selecting the country of interest from a drop-down list (Figure 1). The Web site is available to the public and is free of charge. Over the past year (during its beta phase), we have recorded over 6,000 visits to the site, with a steady increase in traffic over time.

## Implications for Diagnosis of TB

While novel diagnostics have been developed for latent TB infection (LTBI) [15]–[19], the TST continues to be the most widely used diagnostic test worldwide [20]. False positives can occur in BCG-vaccinated individuals, complicating interpretation of test results [21]. However, research suggests the timing of vaccination plays an important role [9],[10]; in a meta-analysis, Farhat et al. found BCG vaccination at infancy has only a minimal effect on TST specificity, particularly if the TST is done more than 10 years after the BCG was administered, whereas BCG later in life or if given more than once led to more frequent, larger, and pronounced TST reactions [21]. The Atlas may help clinicians interpret TST by providing the information necessary to assess whether the TST is a valid diagnostic tool in a particular patient, or when alternative diagnostics may be preferable.

**Figure 3:**
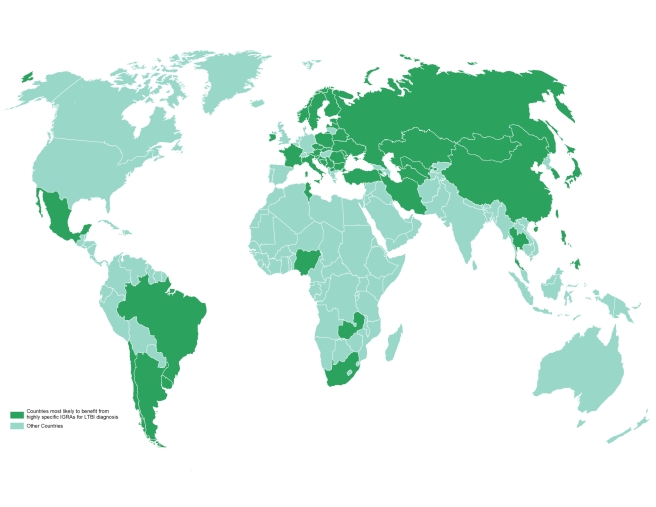
Countries most likely to benefit from highly specific IGRAs for the diagnosis of LTBI. One of the applications of this database is its ability to identify populations where repeated BCG immunizations were administered or where BCG was administered after infancy, as these populations are most likely to benefit from the use of highly specific IGRAs for the diagnosis of LTBI.

Newly available interferon-gamma release assays (IGRAs) are more specific than TST because they are not affected by previous BCG vaccination [19],[22]. Recent meta-analyses show that the specificity of IGRA is high in all populations, and will be of greatest utility in BCG-vaccinated populations [19],[23]. For example, TST may be less specific for LTBI in Japan [24],[25] (Figure 1) or other countries that revaccinate with BCG or have recently ceased revaccination programs (Table 1); in these settings, IGRAs may be more specific than the TST. Conversely, the TST should not suffer from non-specificity in India, for example, where BCG is given once at birth, as has been borne out by research studies [26]–[28]. One of the applications of this database is its ability to identify populations where repeated BCG immunizations were administered or where BCG was administered after infancy, as these populations are most likely to benefit from the use of highly specific IGRAs for the diagnosis of LTBI (Figure 3 and Box 1). Table 3 lists the 22 countries identified by the WHO as representing 80% of the global TB burden, and their respective BCG policies [29]. The six bolded countries have either recommended booster BCGs in the past or currently doing so, while the remaining 16 countries have not recommended booster BCG vaccinations.

**Box 1.** Case Study Using the BCG Atlas.

- A 30-year-old man, born in the Slovak Republic
- Recently arrived in Canada, as a new immigrant
- TST-positive (10 mm), and unremarkable chest X-ray
- No known contact with an active TB case
- No documentation of BCG vaccination status, but has a vague memory of receiving more than one BCG shot

From the BCG Atlas:

- Slovak Republic: Universal BCG vaccinations at birth and BCG boosters at ages 7 and 14 until 2001

→ This individual probably received multiple BCG vaccinations post-infancy, which may seriously compromise specificity of the TST; clinician decides to order an IGRA, and based on a negative IGRA and other clinical factors, clinician decides against recommending isoniazid preventative therapy (IPT).

**Table 3:** 22 High TB burden countries and their respective BCG policies and practices (in order of highest to lowest).

Country	Current BCG Vaccination	Timing of BCG 1	Booster BCG	Timing of BCG 2	Booster BCG in the past
India	Yes	At birth	No	N/A	No
**China**	**Yes**	**At birth**	**No**	**N/A**	**Yes**
Indonesia	Yes	After birth, within 1 yr	No	N/A	No
Nigeria	Yes	At birth	Yes	5 yrs^a^	N/A
Bangladesh	Yes	After birth, within 1 yr	No	N/A	No
Pakistan	Yes	At birth	No	N/A	No
**South Africa**	**Yes**	**At birth**	**No**	**N/A**	**Yes**
Ethiopia	Yes	At birth	No	N/A	No
**Philippines**	**Yes**	**At birth**	**Yes**	**N/A**	**N/A**
Kenya	Yes	At birth	No	N/A	N/A
Congo, Dem. Rep.	Yes	At birth	Unknown	N/A	N/A
**Russian Federation**	**Yes**	**At birth**	**Yes**	**7–14 yrs**	**N/A**
Vietnam	Yes	At birth	No	N/A	No
Tanzania	Yes	At birth	No	N/A	No
**Brazil**	**Yes**	**At birth**	**No**	**N/A**	**Yes**
Uganda	Yes	At birth	Unknown	N/A	N/A
**Thailand**	**Yes**	**At birth**	**No**	**N/A**	**Yes**
Mozambique	Yes	At birth	Unknown	N/A	N/A
Myanmar	Yes	After birth, within 1 yr: 6, 10, 14 weeks	N/A	N/A	
Zimbabwe	Yes	At birth	Unknown	N/A	N/A
Cambodia	Yes	At birth	Unknown	N/A	N/A
Afghanistan	Yes	At birth	Unknown	N/A	N/A

Bolded rows identify countries likely to benefit from IGRAs due to booster BCGs currently or in the past.

a But not yet incorporated into the immunization schedule.

Along with novel approved diagnostics such as the IGRAs, there exist other novel diagnostics in research and development stages that use antigens that vary across BCG strains. These include, for example, the MPB64 patch test and serological tests for the diagnosis of TB [30]–[32]. The BCG policies and practices of a particular country may influence the use and utility of these tests in the future.

## Implications for Immunization Strategies

Recently there has been renewed interest in developing novel vaccines for TB. According to the Global Plan to Stop TB, 2006–2015, “effective TB vaccines will be an essential component of any strategy to eliminate tuberculosis (TB) by 2050” [33]. In 2009, at least six different vaccine candidates completed Phase I clinical trials, and three are currently in Phase II [18]. Novel vaccine candidates include both live and sub-unit vaccines. Many employ a heterologous “prime-boost” strategy that complements the existing immune response to BCG. Either the existing BCG or a new recombinant BCG is administered first, and then the new vaccine serves as a “booster”. Different vaccines are being developed that could be administered in infants and young children pre-exposure, and others as adjuvants to chemotherapy post-exposure. Given that novel vaccines may work to complement the existing BCG, it may be relevant to know what previous BCG vaccination individuals have had, how many and at what ages prior to administering novel “booster vaccines”. Similarly, we may be concerned that antigens from the primary vaccination with BCG may affect the booster vaccine. Therefore, in countries where revaccination with BCG was practiced, we might expect higher rates of Koch response, or delayed hypersensitivity response.

In 2007, the WHO revised its policy on BCG vaccination of children with HIV, making HIV infection in infants a full contra-indication for BCG vaccination, even in settings highly endemic for TB [34]. In 2008, the IUATLD BCG Working Group published a consensus statement supporting the revised WHO BCG vaccination policy, but recommended that current universal BCG immunization of infants continue in countries highly endemic for TB until countries have programs in place for implementing selective deferral of BCG vaccination in infants exposed to HIV [35]. As countries respond to these new global recommendations, changes in vaccination policies because of the HIV epidemic should be captured in future updates of the Atlas.

## Conclusions

Despite nearly a century of use, the BCG vaccine continues to be controversial, and policies and practices vary widely across the world. Many countries have experienced major changes in regards to revaccination over the past 20 years. The BCG World Atlas: A Database of Global BCG Vaccination Policy and Practices is an interactive Web site that attempts to provide the clinician, researcher, and pubic health practitioner alike with resources and information necessary to interpret current and novel TB diagnostics and conduct fruitful research on novel vaccines. Most critically, this is a useful resource for the TB community and is publicly available free of charge through an easy-to-use Web site.

## Abbreviations

BCG: Bacille Calmette-Guérin
IGRA: interferon-gamma release assay
LTBI: latent tuberculosis infection
TB: tuberculosis
TST: tuberculin skin test
WHO: World Health Organization
